# Prevention of Diabetes in Overweight/Obese Adults through Traditional Chinese Patent Medicine: Study Protocol for a Prospective Cohort Study

**DOI:** 10.1155/2021/6006802

**Published:** 2021-11-08

**Authors:** Bing Pang, Yue-Ying Zhang, Hua-Jie Hu, Ye Sun, Ai-Mei Cao, Chun-Qian Lu, Wei-Hua Zhang, Qing Ni

**Affiliations:** ^1^Department of Endocrinology, Guang' Anmen Hospital of China Academy of Chinese Medical Sciences, Beijing, China; ^2^Department of Endocrinology, Beijing Shunyi District Hospital of Chinese Medicine, Beijing, China; ^3^Department of Endocrinology, Beijing Pinggu District Hospital of Chinese Medicine, Beijing, China; ^4^Department of Endocrinology, Beijing Changping District Hospital of Chinese Medicine, Beijing, China; ^5^Department of Endocrinology, Beijing Miyun District Hospital of Chinese Medicine, Beijing, China; ^6^Department of Endocrinology, Beijing Shijitan Hospital, Capital Medical University, Beijing, China

## Abstract

*Background*. Early intervention in prediabetes can prevent or delay the incidence of type 2 diabetes mellitus (T2DM). Traditional Chinese patent medicine (TCPM) is widely used in China to prevent T2DM. This study aims to evaluate the efficacy and safety of TCPMs for preventing T2DM. *Method/Design*. This study is a multicenter, cohort study with two arms. A total of 600 participants will be recruited. The participants will be divided into either intervention or control groups according to their own desire, and the exposure factor is the application of TCPMs. All participants will be encouraged to lead a healthy lifestyle, and the intervention group also used TCPMs based on syndrome differentiation. Incident diabetes and normalization of blood glucose are indexes of end point. Safety assessments and adverse event monitoring will also be conducted. The treatment duration is set for 24 weeks, and we will follow-up for another 2 years. *Discussion*. This trial may provide initial evidence regarding the efficacy and safety of TCPMs plus lifestyle intervention (LI) compared to LI alone for preventing T2DM and provide a comprehensive intervention plans that choose suitable TCPMs for diabetes prevention according to syndrome differentiation. *Trial Registration Number.* Chinese Clinical Trial Registry ID: ChiCTR1900023541, registered on 1 Jun 2019. The version identifier is 2018121702.

## 1. Background

Type 2 diabetes mellitus (T2DM) and its precursor prediabetes are growing public health concerns. In China, it is estimated that one-third of the adult population have prediabetes, and experts have projected that more than 470 million people will have prediabetes by 2030 [1, 2]. Prediabetes is characterized by both mild impaired fasting blood glucose (IFG) and/or impaired glucose tolerance (IGT). People with IGT show normal fasting plasma glucose values (FBG less than 7.0 mmol/L) and abnormal two-hour postprandial blood glucose values (2h PG 7.8 to 11.0 mmol/L). People with IFG only demonstrate an abnormal FBG values (FBG 6.1 to 6.9 mmol/L; 2h PG less than 7.8 mmol/L, if measured). Prediabetes perpetuates pancreatic *β*-cell dysfunction and nearly always precedes T2DM. Furthermore, prediabetes and T2DM often accompany overweight/obesity and dyslipidemia, which are responsible for increased risk of all-cause mortality, cardiovascular disease, and stroke [3]. Therefore, the prevention of T2DM in prediabetic patients is a topic of importance. Guidelines for the prevention and control of T2DM in China recommend that lifestyle interventions and weight loss can prevent prediabetes progress to T2DM [4]. However, prediabetic individuals feel difficult to change their lifestyle, and the challenges also posed in successful implementation and maintenance of certain lifestyle modifications. The pharmacological options recommended in the guidelines for prediabetes are limited.

Currently, there are more than 100 TCPMs used for preventing and treating T2DM, and several systematic reviews have demonstrated that TCPMs combined with moderate lifestyle modification may reduce the risk of progression to T2DM and increase the possibility of regression toward normoglycemia [5, 6]. TCPMs contain various active ingredients, which possess different efficacies and therapeutic principles. In the process of TCM clinical practice, a comprehensive analysis of symptoms and signs is conducted which has implications for determining the cause, nature, and location of the illness and the patient's physical condition and eventually deciding which kinds of TCPMs they should use. Here, our research team will implement a multicenter, prospective cohort study to investigate the efficacy and safety of TCPMs in the prevention of T2DM on the basis of syndrome differentiation and treatment. This work describes the methodology and specific details underlying the study.

## 2. Methods

### 2.1. Trial Design

This study is a multicenter cohort study with two arms. A total of 600 participants will be recruited. The participants will be divided into either intervention or control groups according to their own desire, and the exposure factor is the application of TCPMs. All participants will be encouraged to lead a healthy lifestyle, and the intervention group also used TCPMs based on syndrome differentiation. Incident diabetes and normalization of blood glucose are indexes of end point. Safety assessments and adverse event monitoring will also be conducted. The treatment duration is set for 24 weeks, and we will follow-up for another 2 years.

### 2.2. Ethics, Consent, and Permissions

**Figure 1:**
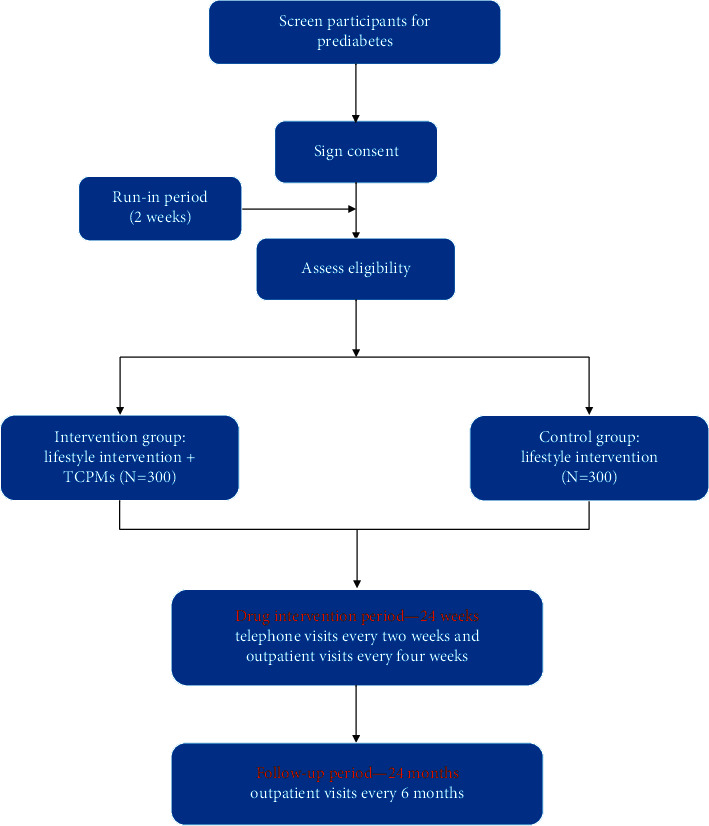
Flow diagram of enrollment, intervention, and assessments.

Approval of protocol and informed consent forms (CRFs) by the local institutional review board was obtained at Guang'anmen Hospital of the China Academy of Chinese Medical Sciences (2019-011-KY). The trial protocol registration number is as follows: Chinese Clinical Trial Registry no. ChiCTR1900023541, and any important changes in the protocol will be reflected there. The trial will be conducted in accordance with the principles of the Declaration of Helsinki (2013 version). When we design the protocol of this study, we refer to the statement of Consolidated Standards of Reporting Trials (CONSORT) and CONSORT Extension for Chinese Herbal Medicine Formulas [7]. We will distribute the CRFs to all participants before enrollment, which will be helpful of understanding the content of these trials and the benefit and harm of this trial. The trial will be completed according to the Standard Protocol Items: Recommendations for Intervention Trials (SPIRIT) guidelines [8]. The trial flow diagram is illustrated in Figure 1.

### 2.3. Study Population and Recruitment

A total of 600 participants will be recruited from six hospitals in mainland China, including Guang'anmen Hospital of the China Academy of Chinese Medical Sciences, Beijing Shijitan Hospital, Capital Medical University, Beijing Changping District Hospital of Chinese Medicine, Beijing Shunyi District Hospital of Chinese Medicine, Beijing Miyun District Hospital of Chinese Medicine, and Beijing Pinggu District Hospital of Chinese Medicine. The participants will be divided into either the intervention group or the control group (1 : 1). Participants should be included according to the diagnostic criteria and inclusion criteria and should be refused according to the exclusion criteria. Eligibility criteria are listed as follows.

### 2.4. Eligibility Criteria

#### 2.4.1. Diagnostic Criteria

Impaired glucose tolerance (IGT) is defined according to the 1999 WHO criteria: FBG <7.0 mmol/L or 2h PBG ≥7.8 and <11.1 mmol/L.

#### 2.4.2. TCM Syndrome Pattern Differentiation

TCM syndrome pattern differentiation is referred to the Diabetes Evidence-based Clinical Practice Guideline in TCM [9] and guidelines delineated in the Clinical Research of New Investigational Drugs in Traditional Chinese Medicine [10].

- Syndrome of dampness-heat accumulation in the spleen: dry mouth and thirst, sweet taste in the mouth, abdominal distension, the subjective sensation of heaviness of the body with difficult movement, and yellow urine, and patients usually have a red tongue with yellow, thick, and dry coating and a rapid strong pulse
- Syndrome of spleen deficiency and phlegm obstruction: overweight or obesity, abdominal obesity, lack of strength, poor appetite, loose stool, bland taste or sticky and greasy sensation in the mouth, pale scalloped tongue, white and greasy coating, and soggy and moderate pulse
- Syndrome of liver depression and qi stagnation: medium or thin shape, dry mouth and thirst, depressed mood frequent sighing, nervousness, distention, fullness in the chest and flank, dry stool, light red tongue, enlarged tongue body, white and thin coating, and stringy pulse
- Qi-yin dual insufficiency syndrome: emaciation of the body, fatigue spirit and lack of strength, dry throat and mouth, thirst even with a large intake of fluid, feeling vexing heat in the five centers (chest, palms, and soles), spontaneous and night sweats, shortness of breath and reluctance to speak, palpitations, insomnia, a red tongue with scant liquids and thin white dry tongue coating, and a thread rapid pulse

#### 2.4.3. Inclusion Criteria

1. Participants should be between 18 and 70 years of age
2. Participants should meet the diagnostic criteria for IGT
3. After a month of routine diet and exercise intervention, participants should meet the diagnostic criteria for IGT
4. Overweight or obesity, body mass index (BMI) ≥  24 kg/m^2^
5. According to syndrome pattern differentiation referred to the Diabetes Evidence-based Clinical Practice Guideline in TCM and guidelines delineated in the Clinical Research of New Investigational Drugs in Traditional Chinese Medicine
6. Participants should sign informed consent forms

#### 2.4.4. Exclusion Criteria

1. Participants who had acute cardiovascular events, myocardial infarction, or stroke within the past 6 months
2. Participants with serum transaminase level two times higher than the upper limit of normal value and serum creatinine two times higher than the upper limit of normal value
3. Participants whose blood glucose rise is caused by a stress state or secondary reason
4. Participants with psychiatric conditions
5. Pregnant, lactating women, or having a history of drug allergy
6. Participants with severe cardiovascular and respiratory disease or severe complications of the liver, kidney, brain, or other primary diseases
7. Participants who suffered acute infections and severe infections
8. Participants who have used hypoglycemic drugs in the past 3 months

#### 2.4.5. Suspension Criteria

1. Participants who experience severe disease or physiological changes, who are not suitable for further study
2. Participants whose blood glucose increased obviously or having severe or frequent hypoglycemia should be withdrawn from the study, and the effect will be judged as invalid, which is a complete case
3. An allergic reaction that is clearly associated with the study drug
4. Adverse symptoms or signs and abnormal examination results occur that are clearly related to intake of the study drug
5. Participants who show poor drug compliance, such as failure to take at least 80% and no more than 120% of the prescribed amount of medicine
6. Women who develop pregnancy during the study
7. Participants who are reluctant to continue this study
8. National laws, ministry of science and technology, or other authorities decide to terminate the study

#### 2.4.6. Withdrawal Criteria

1. Participants who privately cease to accept medical treatment or conduct testing or who become impossible to contact are also considered withdrawn
2. Participants who cannot continue because of poor clinical efficacy, intolerance due to adverse reactions, economic reasons, or moves

### 2.5. Interventions

The participants will be divided into either the intervention group or the control group (1 : 1). The control groups will conduct lifestyle intervention alone, and the intervention group will use TCPMs on the basis of lifestyle intervention.

#### 2.5.1. Lifestyle Intervention

The guidelines for the prevention and treatment of type 2 diabetes (2017 edition) suggest that subjects with abnormal glucose metabolism should reduce their risk of T2DM through controlling diet and strengthening exercise. Lifestyle intervention will be implemented by medical staff, and a health management application will be used. Firstly, healthy lifestyle education will be developed every 2 weeks. The aim of the education is to promote changes in lifestyle behaviors strongly associated to overweight/obesity and the risk of T2DM and to provide personalized health behavior guidance for different target groups. Secondly, the trial will advocate a healthy reasonable match of calories, carbohydrates and protein, and low-salt low-fat and low-sugar diet. The staple food is suggested to be quantitative with the combination of coarse food grain and wheat flour and rice, the daily consumption of vegetables should be enhanced, regular intake of fish and poultry is suggested, appropriate intake of eggs and livestock meat and limited intake of processed meats is suggested, and the intake of sugar, sugar-sweetened beverages, and energy-dense foods is suggested to be reduced. Thirdly, physical activity of moderate intensity, such as brisk walking, swimming, tai chi, and eight-section brocade, is engaged for at least 150 minutes per week. Through the lifestyle interventions mentioned above, the goal for overweight/obese participants is 5–10% of weight reduction in 6–8 months.

#### 2.5.2. Syndrome Differentiation of Traditional Chinese Patent Medicine

The intervention group will use TCPMs based on syndrome differentiation. If the syndrome belongs to dampness-heat accumulation in the spleen, it should be treated with JinQi-Jiangtang tablet (3 tablets, three times per day). If the syndrome belongs to spleen deficiency and phlegm obstruction, it should be treated with Shenzhu Tiaopi granule (2 bags, three times per day). If the syndrome belongs to liver depression and qi stagnation, it should be treated with Yue Ju pills (1 bag, three times per day). If the syndrome belongs to qi-yin dual insufficiency, it should be treated with Qizhi jiangtang capsule (2.5 g three times per day).

#### 2.5.3. Concomitant Treatment

All the participants will receive the standard intervention of blood pressure, paying close attention to other cardiovascular risk factors and giving appropriate intervention measures. Moreover, the participants will not change the drugs used to treat chronic diseases.

### 2.6. Outcome Measurement

#### 2.6.1. Indexes of End Point

1. Incident diabetes according to the guideline for the prevention and treatment of type 2 diabetes (2017 edition)
2. Normalization of Blood Glucose, FPG <5.6 mmol/L or 2h PBG <7.8 mmol/L

#### 2.6.2. Primary Outcome

The primary outcome is the changes of fasting plasma glucose (FBG), two-hour postprandial blood glucose (2h PBG), and glycosylated hemoglobin (HbA1c). Blood glucose is evaluated every 4 weeks, and HbA1c is evaluated every 12 weeks.

#### 2.6.3. Secondary Outcomes

Secondary outcomes are listed as the changes of body mass index (BMI), waist perimeter and hip perimeter, waist-hip ratio, blood lipids (total cholesterol (TC), triglyceride (TG), low-density lipoprotein cholesterol (LDL-C), and high-density lipoprotein cholesterol (HDL-C)), blood pressure, carotic artery intima-media thickness, and improvement in TCM symptoms (evaluated by TCM symptom score scale). Blood pressure, carotic artery intima-media thickness, and TCM symptom score are evaluated every 12 weeks, and the remaining indexes are evaluated every 4 weeks.

#### 2.6.4. Safety Assessment

1. Vital signs (body temperature, blood pressure, respiration, and heart rate), electrocardiogram (ECG), routine blood test, routine urine test, and routine stool test are evaluated at baseline and week 24
2. Liver function including alanine aminotransferase (ALT) and aspartate aminotransferase (AST) and renal function, including serum creatinine (SCr) and blood urea nitrogen (BUN), at baseline and week 24

#### 2.6.5. Questionnaires

Lifestyle intervention is measured using the questionnaire. During the period of intervention, the study participants will be asked to fill in a diary describing the intake of staple food and meals consumed every day. In addition, the participants are asked to weigh every week and register the type and duration of physical activity.

### 2.7. Study Visits and Data Collection

The study is divided into two periods, a 24-week drug intervention period and a 24-month follow-up period. After the intervention period commences, telephone visits will take place every 2 weeks and outpatient visits every 4 weeks. When the follow-up period commences, outpatient visits will take place every 6 months. An overview of data capture can be found listed in Table 1.

### 2.8. Adverse Event Monitoring

Any adverse events (AEs), such as subjective discomfort of participants and abnormal laboratory tests, should be treated seriously, analyzed carefully, and addressed immediately. An adverse event report form will be recorded at every visit, including the occurrence time, severity, duration, adopted measure, and transfer. Every AE will be classified as a mild, moderate, or severe AE, and its relationship between AEs and the intervention drugs will be judged: whether the reasonable relationship exists between the time of drug use and the occurrence of suspicious AEs; whether the suspected AEs conform to the known adverse reactions of the drug; whether the suspicious AEs can be explained by the effect of combined drugs, the clinical condition of patients, or the effect of other therapies; whether the suspected AEs will disappear or alleviate after drug withdrawal or dosage reduction; and whether the same AEs will occur again after reexposure to intervention drugs. Severe AEs will be reported to the principal investigator and the Ethics Committee immediately, and the “Severe Adverse Event (SAE)” form is filled out. The severe adverse events should also be reported to the safety supervision division of the State Food and Drug Administration within 24 hours. All AEs must be treated until properly resolved, retested in 24 hours, 7 days, and 14 days according to the situation, informed the clinical supervisor of the results, and tracked until they are stable.

### 2.9. Data Management and Document Conservation

The researchers should document the data into the case report form (CRF) timely, completely, correctly, and clearly according to original data. The clinical supervisor will confirm that all the CRFs are filled correctly and completely and consistent with the original data. The errors and omissions must correct them in time, the original records should be kept clear and visible when modifying, and the correction should be signed and dated by the researcher. After checked and signed by the supervisor, the CRFs will be sent to the trial data administrator. The data administrator will check the data again before data entry. If any problem is found, they will notify the supervisor and ask the researcher to respond. The exchange between them should be kept in the form of a query table. The data administrator also should understand the content and coding of each item before data entry. The data entry personnel should input the data twice. After data entry, the quality and *l* logicality will be checked. The documents will be requested to conserve by every unit after the study. Researchers should preserve the documents of the clinical trial for five years.

### 2.10. Sample Size Calculation

According to previously published research [11], the annual conversion rate of IGT into T2DM was 1.5% in the TCM group and 10% in the control group. We predicted that the annual conversion rate of IGT into T2DM was 5.7% in the TCM comprehensive intervention group. Therefore, assuming that *α* = 0.05 and *β* = 0.1 according to the one-sided test, u*α* = 1.645 and u*β* = 1.282, and the data will be placed in the formula to obtain *n* = 299.94. Therefore, each group needs more than 300 participants, for a total of 600 participants.

### 2.11. Statistical Analysis

Three datasets will be used for statistical analysis, including a full-analysis set (FAS), per-protocol analysis set (PPS), and safety analysis set (SS). The FAS is the dataset composed of participants who have at least one postmedication evaluation data. The last observation carried forward (LOCF) method will be used for the missing data supplement. The PPS is the dataset composed of participants who meet the inclusion criteria, show good compliance, and complete the treatment plan. The SS is the dataset of all participants randomized to study, and safety record will be assessed, including adverse events and laboratory test results. All statistical tests are two-sided tests, and *P* < 0.05 will be considered statistically significant. Enumeration data will be expressed as frequency and analyzed using the chi-square test or Wilcoxon rank sum test. Measurement data will be described by means, standard deviation, maximum value, and minimum value and compared between groups using the group *t*-test, Fisher's exact test, or Wilcoxon rank sum test. Statistical analysis will be completed by the third-party statisticians.

### 2.12. Quality Control of Study

#### 2.12.1. Research Process Management

The clinical supervisor will regularly inspect to ensure that the process of study is in accord with the protocol and standard practice and confirm that all the CRFs are filled correctly and completely and consistent with the original data. The researchers should record the contents accurately and carefully according to the requirements of CRFs. All the results in clinical trials should be verified to ensure that derive from original data.

#### 2.12.2. Measures for Consistency of Observation

Researchers in this trial will be trained based on a standardized operation practice (SOP) manual and possess the qualifications to carry it on. All research centers will establish uniform SOP and quality control sequence. If necessary, the research centers will hold interim meetings to check the preliminary work, summarize the problems, and propose the solution.

#### 2.12.3. Measures for Compliance of Participants

Researchers should distribute the research drugs to participants in time and supervise the use of drugs. Participants with poor compliance should strengthen the follow-up. Participants are required to bring the remaining drugs distributed last time in order to inspect the drug compliance.

(1) $$\begin{array}{c} \text{Drug compliance}=\left[\frac{\left(\text{dispensing}-\text{remaining}\right)}{\text{dispensing}}\right]\times 100\%. \end{array}$$

## 3. Discussion

In the present clinical practice in China, prevention of T2DM mainly depends on the improvements in unhealthy lifestyles, which is hard to persist in. Several large-scale trials have demonstrated that use of some hypoglycemic drugs can decrease the incidence of diabetes to various degrees in those with prediabetes [12–15], though none are approved by the U.S. Food and Drug Administration, specifically for diabetes prevention. The cost, adverse reactions, tolerance, and other factors are also needed to be considered [4, 16]. TCM may hold a promise in the prevention of T2DM because of its characteristics of “treat disease before it arises,” which include strengthening the body constitution and regulating the high-risk factors leading to diabetes; in addition, TCM could also control the development of T2DM. This cognition corresponds with the thought of the prevention of T2DM. Prediabetes may fall under the TCM patterns of “spleen pyretic abundance,” etc. [17]. The main pathogenesis lies in spleen and stomach congestion, dampness-heat accumulation in the spleen, spleen deficiency and phlegm obstruction, liver depression and qi stagnation, and qi-yin dual insufficiency [9, 10]. In China, many prediabetic patients are more likely to choose the complementary TCPMs. Postmarketing TCPMs possess steady drug composition and defined dosage, which is convenient to observe the efficacy. In recent years, some randomized controlled trials (RCTs) have proved that TCPMs alone or TCPMs combined with lifestyle modification could be an effective intervention for the preventative treatment of T2DM [18–20]. Now, we aim to implement a clinical trial to observe the efficacy of TCPMs in the prevention of T2DM in Chinese subjects. The strength of our trial is that the trial design is a prospective cohort study, not an RCT, and the choice of intervention drugs in our trials is based on syndrome differentiation and treatment, which are more in accord with the clinical practice of TCM. The treatment duration is 6 months, and follow-up is continued for another two years, which is to observe the short-term and long-term effects of TCPMs. As to outcome assessment, we emphasize for incident diabetes and normalization of blood glucose, improvement of symptoms is also important for prediabetic patients to increase the quality of life.

We hypothesize that the prediabetic patients will benefit from TCPMs. If successful, this trial may provide comprehensive intervention plans that choose suitable TCPMs for diabetes prevention according to syndrome differentiation. Although clinical trials are strictly designed, there might also be potential limitations. Firstly, there is no placebo in the control group. Secondly, the long treatment and follow-up period may lead to poor patient compliance, which might greatly influence the therapeutic effect. More effort should be made to find a solution in future trials.

## Figures and Tables

**Table 1:** Schedule of enrollment, allocation, visits, and assessments.

Time of visits	Enrolment	Allocation	Drug intervention period				Follow-up period			
	−2w	0w	4w, 8w	12w	16w, 20w	24w	12 m	18 m	24 m	30 m
*Collect basic medical history*										
Sign informed consent form	√									
Determine inclusion and exclusion criteria	√	√								
Determine withdrawal criteria	√	√	√	√	√	√	√	√	√	√
Fill in general information	√	√								
History of prediabetes and treatment	√									
Comorbidity and symptom	√									
Physical examination and consultation	√	√	√	√	√	√	√	√	√	√
Concomitant medication	√	√	√	√	√	√	√	√	√	√

*Efficacy assessment*										
Glycosylated hemoglobin		√		√		√	√	√	√	√
Fasting plasma glucose		√	√	√	√	√	√	√	√	√
2-hour postprandial blood glucose		√	√	√	√	√	√	√	√	√
Blood lipids		√		√		√	√	√	√	√
Carotic artery intima-media thickness		√		√		√	√	√	√	√
BMI, waist perimeter and hip perimeter, waist-hip ratio, and blood pressure		√	√	√	√	√	√	√	√	√
TCM symptom score		√		√		√	√	√	√	√

*Safety assessment*										
Vital signs		√				√	√	√	√	√
Routine blood, urine, and stool examination		√				√	√	√	√	√
Liver function		√				√	√	√	√	√
Renal function		√				√	√	√	√	√
Electrocardiogram		√				√	√	√	√	√
Hypoglycemic episodes			√	√	√	√	√	√	√	√
Adverse events			√	√	√	√	√	√	√	√

*Other work*										
Record of TCPMs		√	√	√	√	√	√	√	√	√
Research summary						√				√

## Data Availability

Once the main findings of the project are published, the trial steering committee will review all requests for data before access is granted. If appropriate, the anonymized data and associated documentation will be made available to users under a data-sharing agreement.
